# How and Why We Treat and Fill Root-Canals—Our Way

**Published:** 1897-08

**Authors:** W. A. Mills

**Affiliations:** Baltimore, Md.


ARTICLE VI.

### HOW AND WHY WE TREAT AND FILL ROOT-CANALS—OUR WAY.

BY W. A. MILLS, D. D. S , BALTIMORE, MD.

### HOW WE DO IT.

If the root-canal is one from which we have removed the pulp, after having destroyed its vitality with arsenious acid, using the following formula :

℞ Arsenious acid, - grs. xv
Acetate of Morphia, - grs. v

M et. Add sufficient wood creosote to make thin paste; afterwards add enough powdered alum to make a stiff paste.

Or, as we sometimes do, devitalize with a red-hot instrument; again, a straight drill rotated rapidly, producing friction enough to accomplish the desired results.

We swab out the root canal with a saturated solution of tannic acid in glycerol, after which we heat a tiny piece of gutta-percha, and pass it gently to the apex of the root; then we fill the root-canal with any of the filling material, preferably cement. This treatment we give all root-canals where we can reach the apical foramen; and where we cannot, (as instanced in contracted or tortuous root-canals,) we force the tannic acid solution into the inaccessible pulp tissues, after which we fill in the same manner as in the accessible cases. In these cases no systemic conditions are considered.

When the pulp has died from any cause otherwise than that intentionally produced, we proceed, viz., at the first treatment we remove all carious and liquid matter from the pulp chamber, being cautious not to enter the root-canal.

We then saturate a pledget of absorbent cotton with Marchand's peroxid of hydrogen; place it loosely in the pulp chamber; selecting an old steel instrument with sufficient face at the point to fill, or nearly fill the entrance to the pulp chamber, we heat it red-hot and place it upon the pledget of absorbed cotton.

Immediately steam is generated, oxygen is set free, both filling the root-canal with considerable pressure; this is repeated three or four times, or until the patient says heat and pressure are felt within the tissues, outside of the apical foramen.

We then cleanse the root-canal, after which we saturate a roll of absorbent cotton with a solution of equal parts of the tinctures of iodin and aconite, or tincture of iodin alone, and work it into the root-canal, behind this we pack tight a pledget of cotton; fill tooth with temporary filling and dismiss the patient for a few days.

When patient returns, if no inflammatory manifestations have developed, we remove all temporary work, swab out the root canal with the tannic acid solution, and fill with pink gutta percha. In these cases all systemic conditions are taken into consideration, more especially those of tuberculous diathesis.

In cases where there is a fistulous opening, we treat more heroically and fill immediately. In all cases after filling permanently, we paint the gums at the apex of the root with the iodin and aconite mixture.

### WHY WE DO IT.

We use pink gutta-percha because it is one of the best filling materials, and, when placed in contact with living tissues, if, perchance, a little should project a little beyond the apical foramen, it will cause no irritation, as we have often found such cases when extracting for artificial teeth.

The reason why we use it for the whole root-canal in so-called "dead-teeth" is, no one knows (when systemic conditions are not favorable) what moment a sleeping volcano may be awakened. It is the easiest material to remove to give vent to the explosion.

We use the mixture of iodin and aconite, or iodin alone, because they are the two most powerful absorbents we have, and if any irritation of the peridental membrane should be caused by operating, they will remove it promptly.

We use the tannic acid solution because when brought into contact with any pulp tissues left in the root canal, it forms an albuminate of tannin, a compound which is insoluble in any of the fluids of the surrounding tissues, and consequently no disintegration can take place, to cause any after trouble.

We use Marchand's peroxid of hydrogen because, when a red-hot instrument touches the absorbent cotton saturated with it, its water is turned into hot steam; at the same time a large amount of pure oxygen is rapidly liberated, and, being under pressure, permeates not only the root-canal but also the dental tubuli oxidating all septic matter.

Another reason is because if there should be a "blind" abscess the steam and oxygen forcing their way through the apical foramen not only disinfect its contents but also destroys its matrix. The same process bleaches the tooth rapidly. In cases where abscess is persistent, we curette the sac.

Our success has been all we wished in cases of our own devitalization, much being due, we believe, to our manner of applying the arsenious acid paste; with pulpless cases, our failures have been about three out of ten, considering all conditions.—*The Dental News.*

---

